# Stopping the COVID-19 Pandemic: A Review on the Advances of Diagnosis, Treatment, and Control Measures

**DOI:** 10.1155/2020/9121429

**Published:** 2020-10-18

**Authors:** Md. Hasanul Banna Siam, Nahida Hannan Nishat, Ahsan Ahmed, Mohammad Sorowar Hossain

**Affiliations:** ^1^Biomedical Research Foundation (BRF), Dhaka, Bangladesh; ^2^Department of Microbiology, University of Dhaka, Dhaka, Bangladesh; ^3^Dhaka Medical College Hospital, Dhaka, Bangladesh; ^4^School of Environment and Life Science, Independent University Bangladesh (IUB), Dhaka, Bangladesh

## Abstract

With the continued spread of COVID-19 across the world, rapid diagnostic tools, readily available respurposable drugs, and prompt containment measures to control the SARS-CoV-2 infection are of paramount importance. Examples of recent advances in diagnostic tests are CRISPR technology, IgG assay, spike protein detection, and use of artificial intelligence. The gold standard reverse transcription polymerase chain (RT-PCR) has also been upgraded with point-of-care rapid tests. Supportive treatment, mechanical ventilation, and extracorporeal membrane oxygenation (ECMO) remain the primary choice, while therapeutic options include antivirals, antiparasitics, anti-inflammatories, interferon, convalescent plasma, monoclonal antibody, hyperimmunoglobulin, RNAi, and mesenchymal stem cell therapy. Different types of vaccines such as RNA, DNA, and lentiviral, inactivated, and viral vector are in clinical trials. Moreover, rapidly deployable and easy-to-transport innovative vaccine delivery systems are also in development. As countries have started easing down on the lockdown measures, the chance for a second wave of infection demands strict and rational control policies to keep fatalities minimized. An improved understanding of the advances in diagnostic tools, treatments, vaccines, and control measures for COVID-19 can provide references for further research and aid better containment strategies.

## 1. Introduction

In late December 2019, the health officials in China reported of a mysterious pneumonia with no known etiology. Prompt genome analysis revealed that the disease was the cause of a novel coronavirus. As the outbreak continued to spread out at a remarkable pace, the World Health Organization (WHO) had to declare it a pandemic on March 11, 2020. SARS-CoV-2 is the seventh coronavirus to infect humans and the causative agent for COVID-19. Previously, two coronavirus epidemics raised international concern, SARS in 2002–2003 and MERS in 2012. As for the new virus, it is highly infectious and has already killed over 200,000 people with an estimated sCFR (symptomatic case fatality risk) of 1.4% (0.9–2.1%) [1]. By comparison, fatality due to SARS was roughly 10%, whereas, for MERS, it was as high as 35%, making it one of the deadliest human pathogens [2]. However, SARS-CoV-2 has been shown to have much higher human-to-human transmissibility [3]. With the ability to infect people through asymptomatic carriers, it can remain unnoticed and quickly disseminate itself, making the disease containment a confounding public health challenge [4].

WHO recommends nucleic acid amplification test (NAAT) based reverse transcription polymerase chain reaction (RT-PCR) as the primary testing method because of its accuracy and hence it remains the gold standard for COVID-19 detection [5]. However, the technique requires laboratory settings as well as skilled personnel to conduct the test with precision. To scale up the number of tests performed per day, the need for the development of an accurate point-of-care test is of paramount importance. Recently, some antibody-based serological studies provided insights that the number of people having COVID-19 infection could be much higher than what was previously thought.

With overloaded healthcare and an increasing number of infections among the medical personnel, the ultimate way out remains to be in the discovery of an effective vaccine. However, the discovery and development of vaccine or drugs is a lengthy process, and it generally takes around a decade to pass through the entire course. So, repurposing of existing drugs to treat COVID-19 appears as a logical scientific approach. So far, the choices are limited for the treatment of COVID-19 by a lack of specific drugs. From a variety of existing antiviral medications, repurposing the appropriate drug remains as a challenge to overcome. Small-scale studies reported a few drugs to be effective, but later proved to bring no significant difference in clinical outcomes [6–8]. High throughput virtual screening and in vitro studies are underway to look for scopes in both the development and repurposable options of antiviral drugs.

As the virus is moving in a pandemic speed, various control measures have been considered by different regions. Revising the decisions and their respective outcomes can further resolve the challenges in disease containment. In this review, we aimed at summarizing the current literatures to draw a compendium of understanding on the scopes of diagnostics tools, treatments, vaccines, and control measures for COVID-19.

## 2. Diagnostics

### 2.1. The Real Time Reverse Transcriptase Polymerase Chain Reaction (rRT-PCR)

The real time reverse transcriptase polymerase chain reaction (rRT-PCR), or quantitative reverse transcriptase PCR (qRT-PCR), uses nucleic acid amplification test (NAAT) method to convert the viral RNA into cDNA and subsequently amplify the DNA for detection as a definitive confirmatory test [9]. Currently, the targeted viral genes include N, E, S, and RdRP genes. An amplification of each of these genes can be accomplished by the supply of proper forward and reverse primers. One study reported that PCR amplification of the E and SARS coronavirus RdRp genes is 95% sensitive [10]. The RT-PCR based diagnostic is highly sensitive, sequence specific, and useful in the early detection of COVID-19. Since the test accuracy varies depending on the disease stage and viral multiplication, the sensitivity can range from 71 to 98%, whereas the specificity is recorded to be 95% [11].

The RT-PCR has a few disadvantages such as the requirement of strict handling, time consumption, and a minimum initial concentration of RNA. The lowest RNA concentration of SARS-CoV-2 detectable via RT-PCR is found to be 3.8–23 copies/ml [12]. Heat treatment prior to RNA extraction is not recommended as studies suggest that thermal inactivation in samples with low viral loads could result in potential false-negative nucleic acid test [13]. For areas afflicted with COVID-19, a negative PCR result does not imply the absence of the virus since a multitude of factors including viral mutation, PCR inhibition, improper handling of the sample, specimen collection time, low viral RNA, inappropriate shipment, or poor specimen quality can lead to a negative result in an infected individual [14].  Table 1 shows the current diagnostics options for COVID-19.

#### 2.1.1. Types of Specimen

For specimen collection, with regard to upper respiratory specimens, nasopharyngeal and oropharyngeal swab should be considered, and for lower respiratory specimens, sputum and endotracheal aspirate or bronchoalveolar lavage should be considered [5]. Additionally, blood and stool specimen could be collected for clinical inspection. One study reported that the highest rates of positive were found from bronchoalveolar lavage fluid (93%), followed by sputum (72%), nasal swab (63%), fibrobronchoscope brush biopsy (46%), pharyngeal swabs (32%), feces (29%), and blood (1%) [15]. The test result for urine is usually negative, but the presence of SARS-CoV-2 in urine specimen was also described [16]. However, autopsy materials such as lung tissue should be collected in case of deceased patients [5]. To retrospectively detect a case in a surviving patient, serological test is useful as antibodies for past infection are detected.

### 2.2. Development of Point-of-Care Tests

Some point-of-care molecular testing methods have already been approved by emergency use authorization (EUA). For instance, Accula SARS-CoV-2 testing is a qualitative visually read PCR method that provides results in 30 minutes; Abbott ID NOW COVID-19 test uses an isothermal nucleic acid amplification technology to target the RdRp gene, and the positive result can be obtained as quickly as 5 minutes [17, 18].

### 2.3. Immunological Test

#### 2.3.1. Rapid Tests Targeting Viral Proteins

In addition, rapid test design for the detection of viral protein is also in progress. The viral nucleocapsid (N) and spike (S) are the main immunogenic proteins. While nucleocapsid protein is the most abundant and 90% similar to SARS-CoV, viral spike (S) protein is divergent and elicits strong immune response [19]. However, the S1 subunit of spike protein was found to be specific for SARS-CoV-2, but the S2 subunit of spike protein was conserved across coronaviruses [20]. Since many people have antibodies to the four endemic human coronaviruses, targeting specific part of the spike protein could avoid cross-reactivity. Currently, virus culture for detection is not recommend as it is time-consuming and requires biosafety level 3.

#### 2.3.2. Serological Tests Targeting Antibodies

Development of an accurate antibody testing is a major challenge, and as of now, hundreds of trials are ongoing. There are important considerations for antibody testing, (i) timing of the test, (ii) previous infection, (iii) immune status of the individual, and (iv) cross-reaction, which can alter the test result. However, a few antibody-based tests got emergency approval by the FDA, for instance, COVID-19 ELISA IgG Antibody Test from Cellex, which has a sensitivity of 93%, and qSARS-CoV-2 IgG/IgM Rapid Test from Mount Sinai Laboratory with 92.5% sensitivity [21, 22]. The ELISA based antibody test uses the technique of binding assay by way of a recombinant viral antigen that can bind to IgG. Other types of methods include a lateral flow chromatographic immunoassay that qualitatively assesses the presence of an analyte (e.g., antibody) from a patient's whole blood, serum, or plasma specimen. The IgG antibodies bind to the recombinant SARS-CoV-2 antigen-coated gold nanoparticles (AuNP); the rabbit IgG gold-conjugates are used as control that binds to the anti-rabbit antibodies. The assay resembles a lateral flow pregnancy test but detects antibodies instead of a human glycoprotein. Recently, Abbott launched an IgG antibody test that received CE mark with its 99.6% specificity [23]. The Elecsys® AntiSARS-CoV-2 antibody test from Roche received FDA approval that employs in-solution double-antigen sandwich format. The test can detect antibodies in human serum or plasma samples with a specificity greater than 99.8% and sensitivity of 100% after 14 days of PCR confirmation [24].

### 2.4. CRISPR Technology

CRISPR gene editing tool has been employed to construct an accurate, faster, and simple-to-use SARS-CoV-2 detection test. DNA Endonuclease-Targeted CRISPR Trans Reporter (DETECTR) assay is based on CRISPR–Cas12 and it can distinguish SARS-CoV-2 with no cross-reactivity for related coronavirus strains using N gene gRNA within 40 minutes [25]. The result is visualized by using a FAM-biotin reporter molecule and lateral flow strips to capture labeled nucleic acids. Another CRISPR-based POC diagnostic is the SHERLOCK COVID-19 that incorporates a thermostable Cas12b enzyme from *Alicyclobacillus acidiphilus*. The test recently got FDA approval under emergency use authorizations [26]. The test can be conducted by extracting RNA from patient samples and can be read out in less than an hour using a dipstick, without requiring extensive instrumentation. In India, a different approach was taken to build a CRISPR-based tool. The FnCas9 Editor Linked Uniform Detection Assay (FELUDA) used a highly accurate enzymatic readout for detecting nucleotide sequences [27]. The assay is quick to provide output and can be used in rapid diagnosis.

### 2.5. Imaging

Apart from detecting the presence of the virus, chest CT scan can demonstrate the disease status and severity. A recent study suggested that CT scan is more sensitive than the PCR procedure [28]. During the early stage of pneumonia, there are multiple small patchy shadows seen with interstitial changes, which is unusual in the lung periphery [29, 30]. Some cases could develop bilateral multiple ground-glass opacity, infiltrating shadows, and pulmonary consolidation with infrequent pleural effusion [31]. However, CT scans has drawbacks as the mechanism cannot differentiate pneumonia and other pulmonary anomalies from COVID-19 [32].

### 2.6. Artificial Intelligence

Recently, artificial intelligence system based on deep convolutional neural network design was used to detect COVID-19 from chest radiography images [33]. The tool provides quicker result and continues to get better with the addition of more data. Moreover, the machine learning approach shows potential to predict criticality in patients [34]. Some AI inspired mobile application-based tools are in development to preliminary detect suspected COVID-19 patients [35].

### 2.7. Differential Diagnosis

Differential diagnosis is the process by which a single disease or condition is differentiated from those having similar clinical features. Patients with COVID-19 can have coinfection or superimposed infection by other viruses or bacteria simultaneously. Differential diagnosis is therefore important to differentiate SARS-CoV-2 induced infection from other viral or bacterial and mycoplasmal pneumonia. Since a plethora of clinical manifestations is observed in COVID-19, there is an increased chance for incorrect diagnosis. For instance, a COVID-19 patient in Thailand was found to present with fever and rash and was initially mistaken for dengue [36, 37]. In regions with high burden of disease, differential diagnosis based on current symptoms, medical and epidemiological history, and a set of physical examinations will help determine the proper etiology. In case of bacterial pneumonia, there is usually high fever and coughing with thick, blood-tinged mucus or yellowish-greenish sputum with pus. Mycoplasmal pneumonia can also occur in any season, so blood culture or serum antibody determination is helpful for differential diagnosis [35].

## 3. Treatments

### 3.1. Mechanical Ventilation and ECMO

For most patients with mild-to-moderate symptoms, bed rest with supportive treatments including sufficient calorie, water intake and maintaining water electrolyte balance, and homeostasis have shown to provide relief. However, for patients with hypoxia, a noninvasive positive airway pressure ventilator could be used. In case of disease severity, the patient is supported with invasive mechanical ventilation with endotracheal intubation. According to WHO-China joint report, about 25% of severe and critical cases require mechanical ventilation while the remaining 75% can be safely supplemented with oxygen only [38]. With acute respiratory distress syndrome and the failure of mechanical ventilation to improve condition, extracorporeal membrane oxygenation (ECMO) could be performed for critically ill patients. ECMO is a modified cardiopulmonary bypass where the venous blood is drained, oxygen is added, carbon dioxide is removed, and blood is returned to the patient. Studies show that ECMO could reduce mortality in the most severe cases of ARDS [39]. However, ECMO is resource-intensive and requires highly specialized trained professionals to operate as incorrect application can lead to death. Patients who died of COVID-19 showed reduced lymphocyte counts along with a high concentration of IL-6 [40]. Since ECMO application can decrease lymphocytes and increase IL-6 concentration, it is important to consider patient's immune status before providing ECMO support [41].  Table 2 represents the current therapeutic options for COVID-19 treatments.

### 3.2. Repurposable Drugs

#### 3.2.1. Antiviral Drugs

Remdesivir, a prodrug that was previously intended to be used against Ebola virus, is now considered by the WHO as one of the most promising candidates to treat COVID-19 [42, 43]. Recently, the FDA authorized the use of remdesivir under EUA to treat patients as the drug was shown to shorten the recovery time [44]. Remdesivir is an adenosine analogue that targets the viral RdRp and ceases the viral RNA synthesis. The drug is also shown to evade the proofreading capacity by ExoN, thereby inducing irreversible chain termination [45]. The phase-III trial of remdesivir showed that patients treated at the early stage of the disease, that is, prior to the requirement of mechanical ventilation, with a ten-day course of remdesivir, had definitive improvement [46]. However, remdesivir is only available as an IVF (intravenous fluid), and the effectivity of the drug is yet to be established for critically ill ICU patients.

The cocktail of danoprevir and ritonavir is in phase-IV trial indicating to be a promising therapeutic option. Danoprevir is an inhibitor of NS3/4A HCV protease while ritonavir acts to inhibit cellular CYP3A4 to bolster danoprevir's activity. A clinical study conducted in China described eleven patients gaining complete recovery after administering danoprevir boosted with ritonavir [47]. Also, the combination of lopinavir and ritonavir showed improvement in some early studies, but later found to be inefficacious for COVID-19 treatment [47].

Among the other repurposable antiviral drugs, favipiravir is of notable consideration as it acts to selectively inhibit viral RNA polymerase enzyme against a broad range of viruses [48]. The drug has got an emergency approval for treatment in China and shown to provide potent antiviral action against SARS-CoV-2 [49]. Darunavir, a viral protease inhibitor that was originally used against HIV, is now in phase-III (NCT04252274) clinical trial to treat COVID-19. The antiviral drug could be used in combination with cytochrome P450 inhibitors such as cobicistat or ritonavir [50]. Other FDA approved broad spectrum antiviral drugs such as ribavirin and penciclovir are also being tested.

Nafamostat, a potent MERS-CoV inhibitor, was also found active against TMPRSS2-dependent host cell entry of SARS-CoV-2 [51]. To prevent viral fusogenic capacity, nelfinavir mesylate was proposed by one study [52]. Since SARS-CoV-2 can form multinucleated giant cell with extensive syncytial formation in lung tissues, the virus can infect adjacent cells without being detected by neutralizing antibodies. The use of umifenovir, a viral envelope membrane fusion inhibitor, was proposed but it failed to bring any significant positive outcomes in patients, as suggested by one retrospective study from China [53].

#### 3.2.2. Anti-Inflammatory Drugs

Some anti-inflammatory drugs are also made use of to decrease disease severity. For instance, sargramostim is a recombinant GM-CSF that functions as an immunostimulatory agent and augments in innate host defense against pathogens [54]. Among others, tocilizumab is a novel monoclonal antibody that competitively inhibits the binding of interleukin-6 (IL-6) to its receptor (IL-6R), thus reducing the immune hyperactivity [55]. The initial clinical trial of tocilizumab showed effective neutralizing capacity for critically ill patients. Another potential IL-6 inhibitor is sarilumab, currently enrolling patients in trial in Italy, Spain, Germany, France, Canada, and Russia.

Other potential repurposable drugs include steroids as anti-inflammatory agents. Although the use of methylprednisolone in extreme cases such as ARDS indicated a chance of lower mortality, the regular use of corticosteroids is not recommended as the effectiveness is yet to be confirmed [55]. However, janus kinase inhibitor such as ruxolitinib could be a prospective choice as one clinical trial has already progressed to phase-III. However, researchers have also warned of the potential limitation of such drugs to be used for treating COVID-19 [56]. Some ion channel blocker (amiodarone, verapamil), CCR5 antagonist, At1R inhibitor, mTOR inhibitor, sialic acid cleaver, and a wide range of drugs are being tried for as repurposable therapeutic options [57–61].

#### 3.2.3. Antiparasitic Drugs

Previously, preliminary studies indicated that chloroquine/hydroxychloroquine together with azithromycin could potentially reduce the disease severity of COVID-19 patients [62]. In vitro studies also endorsed the effectiveness of chloroquine in blocking human ACE2 receptor [63]. Azithromycin, despite being an antibiotic, could induce potential antiviral effects in epithelial cells [64]. However, more recent studies indicated that the drug is ineffective [6]. The antimalarial drug combined with azithromycin did not show any significant improvement in patients in the United States and France [7, 8].

Recently, an antiparasitic drug suramin was proposed as a possible therapeutic option as it was found to inhibit viral entry [65]. The in vitro study demonstrated that suramin decreased the viral load in Vero E6 and Calu-3 cells. In another cell culture-based study, ivermectin, an antiparasitic drug originally used for the treatment of external parasites and skin conditions, was found to cause ∼5000-fold reduction in SARS-CoV-2 at 48 h [66]. However, authors cautioned about the compassionate use of the drug and recommended a further in-depth evaluation [67].

#### 3.2.4. Interferon Therapy

During SARS outbreak, interferons were used to treat patients, and laboratory experiment showed that IFN-*β* 1a could inhibit SARS-CoV in Vero E-6 cell line [68]. For COVID-19, interferon *β* was proposed as an appropriate treatment choice. A study showed that IFN-I treatment caused significant reduction in viral protein and replication in SARS-CoV-2 [69]. However, the IFN-I treatment did not fully eliminate the virus since SARS-CoV-2 was found to replicate in low levels. This observation could explain in part the reason for paucisymptomatic clinical manifestations in many patients.

### 3.3. Development of New Drugs

The development of novel drug targeting the specific proteins of SARS-CoV-2 is also advancing. One study described deep docking method to identify potential 1000 ligands against the SARS-CoV-2 M^pro^ protein out of 1.3 billion compounds of ZINC15 library [70]. Another study used structure-based drug design protocol to discover potential antiviral leads, for instance, ebselen and thiadiazolidinone-8 (TDZD-8) exhibiting strong antiviral response [71].

Of different viral proteins, 3-chymotrypsin-like protease, papain-like protease, helicase, and RNA-dependent RNA polymerase, spike glycoprotein could be targeted for drug development [72]. Moreover, the already known inhibitors of SARS and MERS could be used as leads to develop newer drugs for COVID-19 [73].

RNAi or RNA interference could be used to construct novel drugs as the technique involves inhibiting gene expression by neutralizing targeted mRNA molecules. The RNAi was extensively explored against SARS-CoV, including the small interfering RNA (siRNA). One study reported that viral replication can be reduced up to 90% using siRNA-based RNAi technology in Vero E6 cells [74].

### 3.4. Plasma Treatment

Convalescent plasma (CP) treatment using blood from recovered COVID-19 patients could be used to aid in recovery for critically ill patients. A considerable number of clinical trials are underway to decide on the safety and efficacy of the treatment, and preliminary results were promising [75]. A study on 10 severely ill adult patients showed that one dose (200 mL) of CP could significantly rise or sustain the concentration of neutralizing antibodies at a high level, contributing to the disappearance of viremia in 7 days [76]. According to the European Commission's recent guidance, the recommended neutralizing antibody titer is 1 : 320 [77]. Despite showing promise, CP treatment may not be available for all due to insufficient supply. Moreover, recovered patients must produce enough neutralizing antibodies to be able to donate blood.

### 3.5. Monoclonal Antibody and Hyperimmunoglobulin

Monoclonal antibodies (mAB) specific to SARS-CoV are also being tried against SARS-CoV-2. One lab experiment reported that the human monoclonal antibody, CR3022, can bind to the receptor binding domain (RBD) of SARS-CoV-2 [78]. Another human mAB 47D11 that binds to the spike glycoprotein of both SARS-CoV and SARS-CoV-2 has also been reported [79].

The development and production of hyperimmunoglobulin (H-Ig) is underway, which could be more potent and easily deployable than convalescent plasma [75]. It contains a fraction of convalescent serum, human IgG. To scale up the production and effectiveness, a recombinant polyclonal H-Ig cocktail, SAB-185, is being developed by SAb Biotherapeutics.

### 3.6. Mesenchymal Stem Cell Therapy

The mesenchymal stem cell (MSC) therapy is also thought to be utilized as they can exert anti-inflammatory and anti-apoptotic effects along with repairing pulmonary epithelial cell damage as well as promoting alveolar fluid damage [80]. Researchers at Lund University, Sweden, developed a lung specific MSC that can reduce lung tissue damage [81]. A pilot study conducted in China showed definitive improvement in seven patients who received MSC treatment [82]. Another study demonstrated that the transplantation of ACE2-mesenchymal stem cells decreased the number of hyperactivated CD4+ T and CD8+ T cells and increased the immunosuppressive cytokine IL-10 [83]. MSCs were shown to be resistant to SARS-CoV-2 infection and had higher expression of anti-inflammatory factors.

## 4. Vaccine

### 4.1. Ongoing Vaccine Trials

The development of vaccine is in progress, but it is estimated that a working safe vaccine will require at least 12–18 months to be available for wider use [84]. There are mainly four types of vaccine strategies used for COVID-19 to provoke immune response: first, using either a weakened or inactivated virus; second, using either replicating or nonreplicating viral vectors, to produce viral proteins inside the body; third, using nucleic-acid-based vaccines, either RNA or DNA, to make copies of viral spike protein using host machinery; fourth, using protein-based vaccines to inject viral protein fragment or virus-like particle inside the body [85]. Around 90 vaccines are undergoing active development and some of them have already started safety trials. The majority of the vaccines are designed to target the viral spike protein as it is the major inducer of neutralizing antibodies [86].  Table 3 summarises the current notable vaccine trials.

The Ad5-nCoV is in phase-II in China and ChAdOx1-nCoV is preparing to enter phase-II trial in the UK. The primary result from the recombinant Ad5-nCoV vaccine expressing the spike glycoprotein showed to stimulate humoral response and the vaccine was found to be safe and tolerable [87]. Other notable vaccine trials include aAPC vaccine, LV-SMENP-DC, INO-4800, and mRNA-1273 [88]. The RNA vaccine is a novel type of vaccine where an RNA of a viral antigenic protein is injected into humans to generate immune response. The mRNA-1273 vaccine was prepared for viral spike protein, and it entered phase 1 clinical trial on March 16, in less than 10 weeks after the viral genetic sequences were published [89]. The use of mRNA vaccine technology provides selective advantage of rapid development over other vaccines due to its inherent functional ability to be readily translated into protein inside the cell. Among other vaccine trial, BCG (Bacille Calmette-Guérin) is being tried. which is originally a vaccine for *tuberculosis*. However, there is no evidence so far to confirm that the BCG vaccine prevents SARS-CoV-2 infection in humans.

### 4.2. Vaccine Delivery Systems

Liquid-based intramuscular needle-and-syringe injections have a few caveats. They are expensive to store and transport; many of them are sensitive to temperature. Some advanced approaches are in active development for COVID-19 such as the Langerhans cell targeted delivery system (LC-TDS) [90]. The LC-TDS employs microneedle patches containing specific ligands embedded in liposomes that are uptaken by the Langerhans cells in the skin, thereby stimulating immune response. The microneedle patches quickly dissolve and allow painless, injury-free administration of vaccines.

Other options that can be explored in vaccine delivery include solid dose vaccine delivery system based on mucosal route to present antigens to mucosa associated lymphoid tissues (MALT). The vaccines currently available via oral route include rotavirus, typhoid, cholera, and poliovirus. Moreover, a live attenuated typhoid vaccine Ty21a is available as orally delivered capsule that can be readily ingested [91]. Fast dissolving tablets (FDT), which are absorbed in the mouth, may be a viable alternative for vaccine administration to address swallowing difficulties in children and elderly subjects [92]. Needle-free powder injection (NFPI) such as ballistic powder injection or intradermal powder injection makes use of dried vaccine coated onto beads. This method could even deliver the vaccine directly into the cytoplasm of the cells [93].

## 5. Control

### 5.1. Protective Measures for Citizens

To curb the crisis, the citizens are advised to follow basic guidelines such as, frequent hand washing, using disinfectants, following cough etiquette, and using facemask. Recent studies suggest that masks could slow down the disease transmission [94]. CDC now recommends using facemask, or at least DIY cloth cover (e.g., 2 layers of cotton fabric, T-shirts, bandanas, or bed sheets) in areas of significant community-based transmission [95]. A study reported that DIY masks made of four-layer kitchen paper and one-layer cloth could block 95.15% of the virus in aerosols, while surgical mask and N95 mask could block up to 97.14% and 99.98%, respectively [96]. However, unless one is sick, it is not recommended to use surgical masks or N95s, which are valuable resource for front-line healthcare workers [95].

COVID-19 is highly infectious, and contact transmission might occur due to touching the mouth, nose, or eyes with contaminated hands. On average, people tend to touch their faces every two and a half minutes, indicating how quickly the virus can make way into a human host [97]. With the ability of SARS-CoV-2 to stay viable on plastic and steel surfaces for up to 3 days, hand hygiene remains to be the effective way to avert the establishment of infection [98]. As an enveloped virus, SARS-CoV-2 is vulnerable to soaps and alcohols that disrupt and dissolve the virus' nearly ordered shell. It is recommended to use soap and water for 20 seconds; and in case of unavailability, hand sanitizers containing at least 60% alcohol may be used. Special attention must be given to the water content while formulating DIY hand sanitizers as water keeps the alcohol from evaporating quickly, allowing the alcohol to encounter the virus for effective inactivation.

### 5.2. Protection for Healthcare Personnel

According to the European standard EN 149 + A1 : 2009, there are three levels of precautions for healthcare workers: (i) contact, (ii) droplet, and (iii) airborne precaution [99]. Medical personnel who work 2 meters away from the patients require contact protection and should wear gloves, mask, and apron. For those who work within 2 meters require droplet precaution and should also use fluid-resistant surgical mask and eyewear (e.g., goggles or a visor), whereas, for health professionals who perform aerosol generating procedures (AGP), they must be prioritized with supplies of gloves, fluid-repellent long sleeved gown, eye protection, and FFP2/3 mask [100, 101]. FFP2, FFP3, and N95 are terms used to refer to high performance filtering masks made of a web of polypropylene microfibers and electrostatic charge. FFP2 and FFP3 can significantly reduce the concentration of hazardous substances up to 10- and 20-fold, respectively [102]. It is important that healthcare workers have training prior to the use of PPE about its proper use and the way of disposal, as improper practice is associated with high rate of infection among the healthcare providers [100].

### 5.3. Lockdown

To contain the virus, a strict lockdown was imposed in Wuhan. Lockdown allows quick suppression of the number of infections facilitating time for the healthcare system to respond to the epidemic with planning and resource mobilization [103]. One study showed that the infection number could be much higher in Mainland China without implementing social distancing [104]. In two weeks, the R0 was reduced from 2.35 to 1.05 in Wuhan, as suggested by a modeling study [105]. A similar effect was observed in the UK as the R0 was found to drop by 73% since the lockdown began [106]. In Singapore, the combination of school closure and social distancing in workplaces showed an efficacy of 99.3% infection prevention [107].

The lockdown strategy and the complete closure of workplaces and termination of domestic and international flights was also argued. One study discussed that the decreasing trend of epidemic in Hubei, China, was possibly not driven by the internal travel ban or lockdown [108]. Another observational study demonstrated that full lockdown policies of France, Italy, Spain, and UK did not have the expected effects.

However, in Brazil, a unique on-off lockdown strategy was undertaken through intermittent relaxation of lockdown [109]. In the Netherlands, an “intelligent lockdown” strategy was followed where schools, museums, and large events remained closed, but many shops and business remained open. People were advised to maintain safe distancing from each other but could move freely [110]. A similar approach was taken by Sweden but with loose restrictions as sports events and large gathering were banned, but business, cafes, and shops remained open for people to take part and lead a regular life [111]. The “Swedish Model” has been both praised and criticized as the number of deaths in Sweden remained higher than its Nordic neighbors.

### 5.4. Tackling the Second Wave

As countries around the world have started easing down on the previously imposed restrictions such as opening up businesses and shops and allowance for travel, there is an increased risk for a second wave of infection, which would be difficult to contain, especially during the winter season. Premature termination of strict measures could potentially lead to a second wave of infection. One modeling study demonstrated that relaxing the lockdown too early would cause the R0 to exceed 1 and spread across China. Epidemiologic disease modeling could play a vital role by predicting the plausible infection rate status. Modern technology such as machine learning could be used to process a large amount of data and generate better models [112].

The continued infection spike across various countries despite lockdown measures indicated that the effectiveness of lockdown depended heavily on the proper testing, contact tracing, quarantining, and imposing strict physical distancing methods in high risk areas [113]. Urban intelligence models built with the help of AI could be developed to employ mass surveillance strategy with data encryption facility for privacy concerns. Antibody-based serological tests should be performed as they show the number of people apparently immune to the virus. The scientific validation and implementation of “immune passport” could potentially reduce infections to exponentially rise.

### 5.5. Fighting Infodemic

Amidst the pandemic, there is an overflow of misinformation regarding the protection and cure from COVID-19. The infodemic of misinformation creates panic and poses a serious threat to public health attempts to contain the virus. Panic buying, false cures, and spread of disinformation amplify the risk of health loss and social disorder. The risk communication team of WHO launched a new platform called WHO Information Network for Epidemics (EPI-WIN) to provide credible information regarding COVID-19 [114]. It is imperative for the governments, media, and concerned citizens to play a viral role in their community by sharing the correct information and raise awareness about the risks of noncompliance to respective guidelines.

## 6. Conclusions

The pandemic of COVID-19 has challenged our existing knowledge, laws, and regulations and forced us to take measures as far as complete lockdown in various parts of the world. The high death toll of COVID-19 has stressed the need for prompt research and dissemination of updated information. This review summarized the scopes and developments of COVID-19 diagnosis tools and therapeutic options and discussed the prevention and control measures considering an apparently upcoming second wave of infection.

While the world is in search for a cure, it is recommended that countries make use of existing scientific tools to develop models to predict community-based outcomes prior to making decisions. Healthcare workers must be supported with supplies and remain updated with the up-to-date knowledge, and citizens must play their role to maintain basic guidelines. At the governmental level, facilitating more testing and contact tracing, providing timely publication of epidemic information, enabling early diagnosis, and delivering supportive treatments for the patients are of utmost importance.

## Figures and Tables

**Table 1:** A comparison of current diagnostic options for COVID-19.

Platform	Specimen/material	Type	Time	Sensitivity (%)	Specificity (%)	Cross-reactivity
NAAT	Nasopharyngeal swab; sputum; bronchoalveolar lavage fluid; blood	RT-PCR	240 mins	71–98	95	No
		CRISPR	40 mins	97	100	No

Serological	Nasopharyngeal swab; blood	Antibody	15–30 mins	92–100	93–100	Yes
		Antigen	15–30 mins	66–92	100	Yes

Imaging	Chest X-ray Chest CT-scan	Radiologist	15–30 mins	74.6	93.8	NA
		AI	<5 mins	84.3	82.8	NA

**Table 2:** Current therapeutic options for COVID-19.

Class	Medication	Mechanism of action	Common use in/against	Trials (*n*)	Phase
Antiviral	Remdesivir	RdRp inhibitor	Ebola	19	Phase-III (NCT04280705)
	Favipiravir	RdRp inhibitor	Influenza	41	Phase-IV (NCT04359615)
	Lopinavir/Ritonavir	Inhibits viral protease	HIV	48	Phase-IV (NCT04252885)
	Danoprevir	Inhibits viral protease	HCV	3	Phase-IV (NCT04291729)
	Nafamostat	Inhibits TMPRSS2 enzyme	Pancreatitis	3	Phase-III (NCT04418128)
	Ribavirin	Inhibits inosine monophosphate dehydrogenase	RSV, HCV	9	Phase-III (NCT04460443)
	Umifenovir	Viral envelope membrane fusion inhibitor	Influenza	4	Phase-IV (NCT04350684)

Anti-inflammatory	Tocilizumab	Humanized IL-6 receptor antibody	Rheumatoid arthritis	55	Phase-IV (ChiCTR2000033705)
	Sarilumab	Humanized IL-6 receptor antibody	Rheumatoid arthritis	19	Phase-III (NCT04327388)
	Sargramostim	Humanized GM-CSF to restore immune homeostasis	Acute myeloid leukemia	3	Phase-IV (NCT04326920)
	Ruxolitinib	Janus kinase 1/2 inhibitor	Myelofibrosis	21	Phase-III (NCT04348071)
	Methylprednisolone	Suppresses host inflammatory responses	Immune suppressor	15	Phase-III (NCT04244591)
	Monoclonal antibody	Binds to RBD of SARS-CoV-2	Wider usage	57	Phase-III (NCT04483960)

Antiparasitic	Chloroquine/hydroxychloroquine	Increases endosomal pH; interferes viral fusion with cell	Malaria	100+	Phase-IV (NCT04466540)
	Ivermectin	Prevents translocation of viral proteins into nucleus	Parasitic infection	32	Phase-IV (NCT04435587)
	Suramin	Inhibits viral replication at early phase	Sleeping sickness	1	(CHICTR2000030029)

Interferon	IFN-*β* 1a	Activates host immune system	Multiple sclerosis	1	Phase-III (IRCT20080901001165N53)

Others	Convalescent plasma	Antibodies target SARS-CoV-2	Wider usage	100+	(NCT04264858)
	Mesenchymal stem Cell	Decreases hyperactivated T cells and increases IL-10	IMID	100+	Phase-III (IRCT20200426047206N2)

RdRp = RNA-dependent RNA polymerase; IMID = immune-mediated inflammatory diseases.

**Table 3:** Notable COVID-19 vaccine candidates in clinical trial.

Platform	Vaccine	Company	Stage	Location
RNA	mRNA 1273	Moderna	Phase-I (NCT04283461)	USA
	BNT 162	Pfizer, BioNTech	Phase-I (NCT04380701)	Germany, USA

DNA	INO 4800	Inovio Pharmaceuticals	Phase-I (NCT04336410)	USA
		Korean Institute of Health	Phase-I	South Korea

Lentiviral	LV-SMENP-DC	Shenzhen Genoimmune Medical Institute	Phase-I (NCT04276896)	China
	aAPC vaccine	Shenzhen Genoimmune Medical Institute	Phase-I (NCT04299724)	China

Inactivated	PiCoVacc	Sinovac BioTech	Phase-I (NCT04352608)	China
	COVID-19 vaccine	Wuhan Institute of Biological Products (Sinopharm)	Phase-I	China

Nonreplicating viral vector	ChAdox1 nCoV-19	University of Oxford	Phase-I (NCT04324606)	UK
	Ad5- nCoV	CanSino Biologics	Phase-II (NCT04341389)	China

Live attenuated	BCG vaccine	Max Planck Institute	Phase-III	Germany
	BCG vaccine	Radboud University	Phase-III	Netherlands
	BCG vaccine	Texas A&M University	Phase-IV	USA

## References

[1] Wu J. T., Leung K., Bushman M. (2020). Estimating clinical severity of COVID-19 from the transmission dynamics in Wuhan, China. *Nature Medicine*.

[2] De Wit E., Van Doremalen N., Falzarano D., Munster V. J. (2016). SARS and MERS: recent insights into emerging coronaviruses. *Nature Reviews Microbiology*.

[3] Sanche S., Lin Y. T., Xu C., Romero-Severson E., Hengartner N., Ke R. (2020). High contagiousness and rapid spread of severe Acute respiratory syndrome coronavirus 2. *Emerging Infectious Diseases*.

[4] Rothe C., Schunk M., Sothmann P. (2020). Transmission of 2019-NCOV infection from an asymptomatic contact in Germany. *New England Journal of Medicine*.

[5] WHO (2020). *Laboratory Testing for Coronavirus Disease 2019 (COVID-19) in Suspected Human Cases. Interim Guidance*.

[6] Mahevas M., Tran V.-T., Roumier M. (2020). No evidence of clinical efficacy of hydroxychloroquine in patients hospitalized for COVID-19 infection with oxygen requirement: results of a study using routinely collected data to emulate a target trial. *MedRxiv*.

[7] Magagnoli J., Narendran S., Pereira F. (2020). Outcomes of hydroxychloroquine usage in United States veterans hospitalized with Covid-19. *MedRxiv*.

[8] Molina J. M., Delaugerre C., Le Goff J. (2020). No evidence of rapid antiviral clearance or clinical benefit with the combination of hydroxychloroquine and azithromycin in patients with severe COVID-19 infection. *Médecine et Maladies Infectieuses*.

[9] Accelerated Emergency Use Authorization (EUA) Summary COVID-19 RT-PCR Test (Laboratory Corporation of America), https://www.fda.gov/media/136151/download

[10] Corman V. M., Landt O., Kaiser M. (2020). Detection of 2019 novel coronavirus (2019-nCoV) by real-time RT-PCR. *Eurosurveillance*.

[11] Watson J., Whiting P. F., Brush J. E. (2020). Interpreting a COVID-19 test result. *BMJ*.

[12] Van Kasteren P. B., Van der Veer B., Van den Brink S. (2020). Comparison of seven commercial RT-PCR diagnostic kits for COVID-19. *Journal of Clinical Virology*.

[13] Pan Y., Long L., Zhang D. (2020). Potential false-negative nucleic acid testing results for severe Acute respiratory syndrome coronavirus 2 from thermal inactivation of samples with low viral loads. *Clinical Chemistry*.

[14] (2020). *False Negatives and Reinfections: The Challenges of SARS-CoV-2 RT-PCR Testing*.

[15] Wang W., Xu Y., Gao R. (2020). Detection of SARS-CoV-2 in different types of clinical specimens. *Journal of the American Medical Association*.

[16] Peng L., Liu J., Xu W. (2020). 2019 novel coronavirus can be detected in urine, blood, anal swabs and oropharyngeal swabs samples. *MedRxiv*.

[17] (2020). *Accula SARS-CoV-2 Diagnostic Test for COVID-19—Mesa Biotech*.

[18] (2020). *ID NOWTM COVID-19-Rapid Point of Care Diagnostics-Abbott*.

[19] Ahmed S. F., Quadeer A. A., McKay M. R. (2020). Preliminary identification of potential vaccine targets for the COVID-19 coronavirus (SARS-CoV-2) based on SARS-CoV immunological studies. *Viruses*.

[20] Okba N. M. A., Müller M. A., Li W. (2020). Severe acute respiratory syndrome coronavirus 2-specific antibody responses in coronavirus disease 2019 patients. *Emerging Infectious Diseases*.

[21] FDA (2020). *COVID-19 ELISA IgG Antibody Test*.

[22] FDA (2020). *qSARS-CoV-2 IgG/IgM Rapid Test*.

[23] Mahase E. (2020). Covid-19: antibody test that claims to be 99% accurate is certified by EU. *BMJ (Clinical Research ed.)*.

[24] Roche https://www.roche.com/media/releases/med-cor-2020-05-03.htm.

[25] Broughton J. P., Deng X., Yu G. (2020). CRISPR–Cas12-based detection of SARS-CoV-2. *Nature Biotechnology*.

[26] Joung J., Ladha A., Saito M. (2020). Point-of-care testing for COVID-19 using SHERLOCK diagnostics. *MedRxiv*.

[27] Azhar M., Phutela R., Ansari A. H. (2020). Rapid, field-deployable nucleobase detection and identification using FnCas9. *BioRxiv*.

[28] Al-Tawfiq J. A., Memish Z. A. (2020). Diagnosis of SARS-CoV-2 infection based on CT scan vs RT-PCR: reflecting on experience from MERS-CoV. *Journal of Hospital Infection*.

[29] Huang C., Wang Y., Li X. (2020). Clinical features of patients infected with 2019 novel coronavirus in Wuhan, China. *The Lancet*.

[30] Chan J. F.-W., Yuan S., Kok K.-H. (2020). A familial cluster of pneumonia associated with the 2019 novel coronavirus indicating person-to-person transmission: a study of a family cluster. *The Lancet*.

[31] Wu D., Wu T., Liu Q., Yang Z. (2020). The SARS-CoV-2 outbreak: what we know. *International Journal of Infectious Diseases*.

[32] Li X., Geng M., Peng Y., Meng L., Lu S. (2020). Molecular immune pathogenesis and diagnosis of COVID-19. *Journal of Pharmaceutical Analysis*.

[33] Wang L., Wong A. (2020). COVID-Net: a tailored deep convolutional neural network design for detection of COVID-19 cases from chest X-Ray images. https://arxiv.org/abs/2003.09871.

[34] Yan L., Zhang H.-T., Xiao Y. (2020). Prediction of criticality in patients with severe Covid-19 infection using three clinical features: a machine learning-based prognostic model with clinical data in Wuhan. *medRxiv*.

[35] Imran A., Posokhova I., Qureshi H. N. (2020). AI4COVID-19: AI enabled preliminary diagnosis for COVID-19 from cough samples via an app. *Informatics in Medicine Unlocked*.

[36] Joob B., Wiwanitkit V. (2020). COVID-19 can present with a rash and be mistaken for dengue. *Journal of the American Academy of Dermatology*.

[37] Henrina J., Cahyo I., Putra S., Lawrensia S., Handoyono Q. F., Cahyadi A. (2020). Coronavirus disease of 2019 : a mimicker of dengue infection?. *SN Comprehensive Clinical Medicine*.

[38] Aylward W., Liang B. (2019). Report of the WHO-China Joint Mission on coronavirus disease 2019 (COVID-19). *WHO-China jt. Mission Coronavirus Dis*.

[39] Goligher E. C., Tomlinson G., Hajage D. (2018). Extracorporeal membrane oxygenation for severe acute respiratory distress syndrome and posterior probability of mortality benefit in a post hoc Bayesian analysis of a randomized clinical trial. *JAMA*.

[40] Ruan Q., Yang K., Wang W., Jiang L., Song J. (2020). Clinical predictors of mortality due to COVID-19 based on an analysis of data of 150 patients from Wuhan, China. *Intensive Care Medicine*.

[41] Henry B. M. (2020). COVID-19, ECMO, and lymphopenia: a word of caution. *The Lancet Respiratory Medicine*.

[42] Malik Y. S., Sircar S., Bhat S. (2020). Emerging novel coronavirus (2019-nCoV)-current scenario, evolutionary perspective based on genome analysis and recent developments. *Veterinary Quarterly*.

[43] World Health Organization (2020). *WHO R&D Blueprint Informal Consultation on Prioritization of Candidate Therapeutic Agents for Use in Novel Coronavirus 2019 Infection*.

[44] (2020). *Coronavirus (COVID-19) Update: FDA Issues Emergency Use Authorization for Potential COVID-19 Treatment*.

[45] Agostini M. L., Andres E. L., Sims A. C. (2018). Coronavirus susceptibility to the antiviral remdesivir (GS-5734) is mediated by the viral polymerase and the proofreading exoribonuclease. *MBio*.

[46] Beigel J. H., Tomashek K. M., Dodd L. E. (2020). Remdesivir for the treatment of covid-19—preliminary report. *New England Journal of Medicine*.

[47] Chen H., Zhang Z., Wang L. (2020). First clinical study using HCV protease inhibitor danoprevir to treat naive and experienced COVID-19 patients. *MedRxiv*.

[48] Delang L., Abdelnabi R., Neyts J. (2018). Favipiravir as a potential countermeasure against neglected and emerging RNA viruses. *Antiviral Research*.

[49] Dong L., Hu S., Gao J. (2020). Discovering drugs to treat coronavirus disease 2019 (COVID-19). *Drug Discoveries & Therapeutics*.

[50] Gasmi A., Noor S., Tippairote T., Dadar M., Menzel A., Bjørklund G. (2020). Individual risk management strategy and potential therapeutic options for the COVID-19 pandemic. *Clinical Immunology*.

[51] Hoffmann M., Schroeder S., Kleine-Weber H., Müller M. A., Drosten C., Pöhlmann S. (2020). Nafamostat mesylate blocks activation of SARS-CoV-2: new treatment option for COVID-19. *Antimicrobial Agents and Chemotherapy*.

[52] Musarrat F., Chouljenko V., Dahal A. (2020). The anti‐HIV drug nelfinavir mesylate (Viracept) is a potent inhibitor of cell fusion caused by the SARSCoV‐2 spike (S) glycoprotein warranting further evaluation as an antiviral against COVID‐19 infections. *Journal of Medical Virology*.

[53] Lian N., Xie H., Lin S., Huang J., Zhao J., Lin Q. (2020). Umifenovir treatment is not associated with improved outcomes in patients with coronavirus disease 2019: a retrospective study. *Clinical Microbiology and Infection*.

[54] Potter H., Boyd T. D., Clarke P., Pelak V. S., Tyler K. L. (2020). Recruiting the innate immune system with GM-CSF to fight viral diseases, including West Nile Virus encephalitis and COVID-19. *F1000Research*.

[55] Sebba A. (2008). Tocilizumab: the first interleukin-6-receptor inhibitor. *American Journal of Health-System Pharmacy*.

[56] Praveen D., Puvvada R. C., Vijey Aanandhi V. (2020). Janus kinase inhibitor baricitinib is not an ideal option for management of COVID-19. *International Journal of Antimicrobial Agents*.

[57] M D. H. F., Nas J., Pinto-Sietsma S.-J. (2020). Rationale and design of the PRAETORIAN-COVID trial: a double-blind, placebo-controlled randomized clinical trial with valsartan for PRevention of Acute rEspiraTORy dIstress syndrome in hospitAlized patieNts with SARS-COV-2 Infection Disease. *American Heart Journal*.

[58] Amiodarone or Verapamil in COVID-19 hospitalized patients with symptoms, https://clinicaltrials.gov/ct2/show/NCT04351763

[59] Efficacy and safety of sirolimus in COVID-19 infection, https://clinicaltrials.gov/ct2/show/NCT04461340

[60] DAS181 for STOP COVID-19, https://clinicaltrials.gov/ct2/show/NCT04354389

[61] Study to Evaluate the Efficacy and Safety of Leronlimab for Mild to Moderate COVID-19, https://clinicaltrials.gov/ct2/show/NCT04343651

[62] Chen J., Liu D., Liu L. (2020). A pilot study of hydroxychloroquine in treatment of patients with common coronavirus disease-19 (COVID-19). *Journal of Zhejiang University (Medicine Science)*.

[63] Wang M., Cao R., Zhang L. (2020). Remdesivir and chloroquine effectively inhibit the recently emerged novel coronavirus (2019-nCoV) in vitro. *Cell Research*.

[64] Schögler A., Kopf B. S., Edwards M. R. (2015). Novel antiviral properties of azithromycin in cystic fibrosis airway epithelial cells. *European Respiratory Journal*.

[65] Benvindo C. S., Thaler M., Tas A. (2020). Suramin inhibits SARS-CoV-2 infection in cell culture by interfering with early steps of the replication cycle. *BioRxiv*.

[66] Caly L., Druce J. D., Catton M. G., Jans D. A., Wagstaff K. M. (2020). The FDA-approved drug ivermectin inhibits the replication of SARS-CoV-2 in vitro. *Antiviral Research*.

[67] Bray M., Rayner C., Noël F., Jans D., Wagstaff K. (2020). Ivermectin and COVID-19: a report in antiviral research, widespread interest, an FDA warning, two letters to the editor and the authors’ responses. *Antiviral Research*.

[68] Hensley L. E., Fritz E. A., Jahrling P. B., Karp C., Huggins J. W., Geisbert T. W. (2004). Interferon-*β* 1a and SARS coronavirus replication. *Emerging Infectious Diseases*.

[69] Lokugamage K. G., Hage A., Schindewolf C., Rajsbaum R., Menachery V. D. (2020). SARS-CoV-2 is sensitive to type I interferon pretreatment. *BioRxiv*.

[70] Ton A. T., Gentile F., Hsing M., Ban F., Cherkasov A. (2020). Rapid identification of potential inhibitors of SARS-CoV-2 main protease by deep docking of 1.3 billion compounds. *Molecular Informatics*.

[71] Jin Z., Du X., Xu Y. (2020). Structure of Mpro from COVID-19 virus and discovery of its inhibitors. *Nature*.

[72] Li G., De Clercq E. (2020). Therapeutic options for the 2019 novel coronavirus (2019-nCoV). *Nature Reviews Drug Discovery*.

[73] Morse J. S., Lalonde T., Xu S., Liu W. R. (2020). Learning from the past: possible urgent prevention and treatment options for severe Acute respiratory infections caused by 2019-nCoV. *ChemBioChem*.

[74] Wu C.-J., Huang H.-W., Liu C.-Y., Hong C.-F., Chan Y.-L. (2005). Inhibition of SARS-CoV replication by siRNA. *Antiviral Research*.

[75] Sheridan C. (2020). Convalescent serum lines up as first-choice treatment for coronavirus. *Nature Biotechnology*.

[76] Duan K., Liu B., Li C. (2020). Effectiveness of convalescent plasma therapy in severe COVID-19 patients. *Proceedings of the National Academy of Sciences of the United States of America*.

[77] European Commision (2020). *An EU Programme of COVID-19 Convalescent Plasma Collection and Transfusion Guidance on Collection, Testing, Processing, Storage, Distribution and Monitored Use*.

[78] Tian X., Li C., Huang A. (2020). Potent binding of 2019 novel coronavirus spike protein by a SARS coronavirus-specific human monoclonal antibody. *Emerging Microbes & Infections*.

[79] Wang C., Li W., Drabek D. (2020). A human monoclonal antibody blocking SARS-CoV-2 infection. *Nature Communication*.

[80] Cao X. (2020). COVID-19: immunopathology and its implications for therapy. *Nature Reviews Immunology*.

[81] (2020). *Can Stem Cells Treat COVID-19?*.

[82] Ankrum J. (2020). Can cell therapies halt cytokine storm in severe COVID-19 patients?. *Science Translational Medicine*.

[83] Leng Z., Zhu R., Hou W. (2020). Transplantation of ACE2- Mesenchymal stem cells improves the outcome of patients with covid-19 pneumonia. *Aging and Disease*.

[84] Klein A. (2020). Drug trials under way. *New Scientist*.

[85] Callaway E. (2020). The race for coronavirus vaccines: a graphical guide. *Nature*.

[86] Dhama K., Sharun K., Tiwari R. (2020). COVID-19, an emerging coronavirus infection: advances and prospects in designing and developing vaccines, immunotherapeutics, and therapeutics. *Human Vaccines & Immunotherapeutics*.

[87] Zhu F.-C., Li Y.-H., Guan X.-H. (2020). Safety, tolerability, and immunogenicity of a recombinant adenovirus type-5 vectored COVID-19 vaccine: a dose-escalation, open-label, non-randomised, first-in-human trial. *Lancet*.

[88] Thanh Le T., Andreadakis Z., Kumar A. (2020). The COVID-19 vaccine development landscape. *Nature Reviews Drug Discovery*.

[89] Lurie N., Saville M., Hatchett R., Halton J. (2020). Developing covid-19 vaccines at pandemic speed. *New England Journal of Medicine*.

[90] (2020). *Corona: Vaccination without a needle?*.

[91] FDA (2013). *Vivotif Package Insert USA-Updated September 2013-Increase of Upper Specification Limit Vivotif® Typhoid Vaccine Live Oral Ty21a*.

[92] Lal M., Priddy S., Bourgeois L. (2013). Development of a fast-dissolving tablet formulation of a live attenuated enterotoxigenic *E. coli* vaccine candidate. *Vaccine*.

[93] Weissmueller N. T., Marsay L., Schiffter H. A. (2017). Alternative vaccine administration by powder injection: needle-free dermal delivery of the glycoconjugate meningococcal group *Y* vaccine. *PLoS One*.

[94] Leung N. H. L., Chu D. K. W., Shiu E. Y. C. (2020). Respiratory virus shedding in exhaled breath and efficacy of face masks. *Nature Medicine*.

[95] (2020). *Recommendation Regarding the Use of Cloth Face Coverings*.

[96] Ma Q. X., Shan H., Zhang H. L., Li G. M., Yang R. M., Chen J. M. (2020). Potential utilities of mask-wearing and instant hand hygiene for fighting SARS-CoV-2. *Journal of Medical Virology*.

[97] Kwok Y. L. A., Gralton J., McLaws M.-L. (2015). Face touching: a frequent habit that has implications for hand hygiene. *American Journal of Infection Control*.

[98] Van Doremalen N., Bushmaker T., Morris D. H. (2020). Aerosol and surface stability of SARS-CoV-2 as compared with SARS-CoV-1. *New England Journal of Medicine*.

[99] BS. EN 149:2001+A1:2009 (2001). *Respiratory Protective Devices. Filtering Half Masks to Protect Against Particles. Requirements, Testing, Marking*.

[100] Cook T. M. (2020). Personal protective equipment during the coronavirus disease (COVID) 2019 pandemic–a narrative review. *Anaesthesia*.

[101] ECDC (2020). *Infection Prevention and Control for COVID-19 in Healthcare Settings-First Update 12 March 2020*.

[102] Gawn J., Clayton M., Makison C., Crook B., Hill H. (2008). Evaluating the protection afforded by surgical masks against influenza bioaerosols. *Health and Safety Executive*.

[103] Musinguzi G., Asamoah B. O. (2020). The science of social distancing and total lock down: does it work? Whom does it benefit?. *Electronic Journal of General Medicine*.

[104] Zhang Y., Jiang B., Yuan J., Tao Y. (2020). The impact of social distancing and epicenter lockdown on the COVID-19 epidemic in mainland China: a data-driven SEIQR model study. *MedRxiv*.

[105] Kucharski A. J., Russell T. W., Diamond C. (2020). Early dynamics of transmission and control of COVID-19: a mathematical modelling study. *The Lancet Infectious Diseases*.

[106] (2020). *Impact of Physical Distance Measures on Transmission in the UK*.

[107] Koo J. R., Cook A. R., Park M. (2020). Interventions to mitigate early spread of SARS-CoV-2 in Singapore: a modelling study. *Lancet Infectious Diseases*.

[108] Ku C.-C., Vincent Ng T.-C., Lin H.-H. (2020). Epidemiological benchmarks of the COVID-19 outbreak control in China after Wuhan’s lockdown: a modelling study with an empirical approach. *SSRN Electronic Journal*.

[109] Tarrataca L., Dias C. M., Haddad D. B., Arruda E. F. (2020). Flattening the curves: on-off lock-down strategies for COVID-19 with an application to Brazi. https://arxiv.org/abs/2004.06916.

[110] (2020). *Life After Lockdown: Dutch Hope “Intelligent” Strategy Pays off*.

[111] Savage M. (2020). *Coronavirus: Has Sweden Got its Science Right?*.

[112] Abhari R. S., Marini M., Chokani N. (2020). COVID-19 epidemic in Switzerland: growth prediction and containment strategy using artificial intelligence and big data. *medRxiv*.

[113] Wilder-Smith A., Freedman D. O. (2020). Isolation, quarantine, social distancing and community containment: pivotal role for old-style public health measures in the novel coronavirus (2019-nCoV) outbreak. *Journal of Travel Medicine*.

[114] Zarocostas J. (2020). How to fight an infodemic. *The Lancet*.

